# Supplementary material for: Disruption of Redox Homeostasis by Alterations in Nitric Oxide Synthase Activity and Tetrahydrobiopterin along with Melanoma Progression
Source: Int J Mol Sci. 2022 May 26;23(11):5979. doi: 10.3390/ijms23115979 (PMC9181279; doi:10.3390/ijms23115979)
Supplement: Supplementary file 1 [file ijms-23-05979-s001.zip › ijms-1718131-supplementary.pdf]

Supplementary Materials

The differential contribution of tetrahydrobiopterin along melanoma progression

Jaqueline Pereira Moura Soares[1,†], Diego Assis Gonçalves[2,3,†], Ricardo Xisto de Sousa[1], Margareth G. Mouro[4], Elisa M. S. Higa[4], Letícia Paulino Sperandio[5], Carolina Moraes Vitoriano[6], Elisa Bachir Santa Rosa[6], Fernanda Oliveira dos Santos[6], Gustavo Nery de Queiroz[6], Roberta Sessa Stilhano Yamaguchi1, Gustavo Pereira[5], Marcelo Yudi Icimoto[7], , Fabiana Henriques Machado de Melo[6,*].

* Correspondence: FHMM: fabihmmelo2020@gmail.com, fabihmmelo@usp.br

† These authors contributed equally to this work and share first authorship

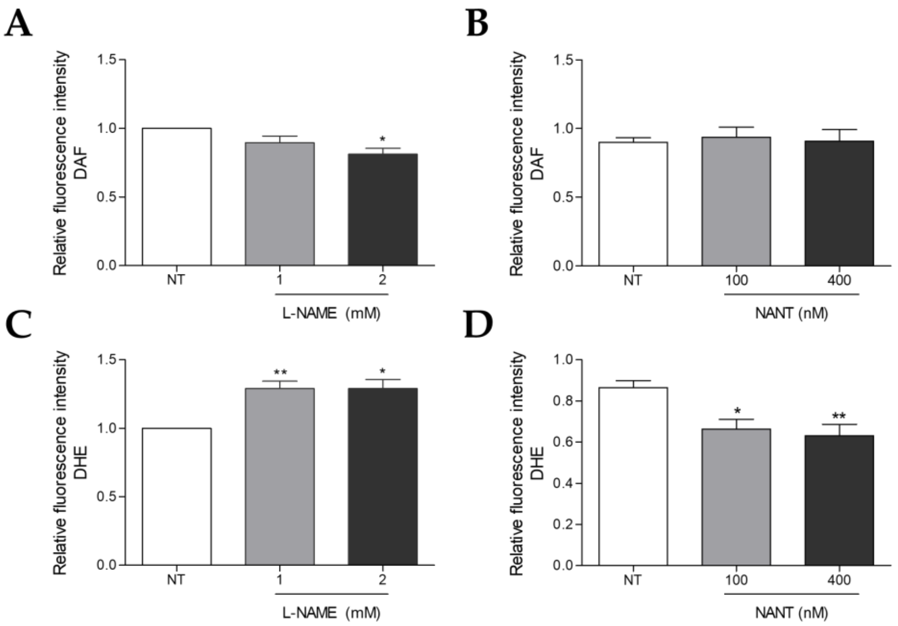

Figure S1. Neuronal nitric oxide synthase is uncoupled in radial growth phase melanoma. WM1552C cells were treated or not for 45 min with 1 and 2 mM L-NAME, an eNOS inhibitor **(A, C)** or 100 and 400 nM NANT, a nNOS inhibitor **(B, D)**.NO amount was evaluated by flow cytometry using DAF **(A,B)** and $O_2^{\bullet -}$ levels using DHE **(C,D)**. Values are reported in the bar graphs and expressed as the means ± S.D. The experiments were performed in triplicate and p values were based on One-Way ANOVA test followed by Bonferroni's post-test *$p<0.05$, **$p<0.01$.

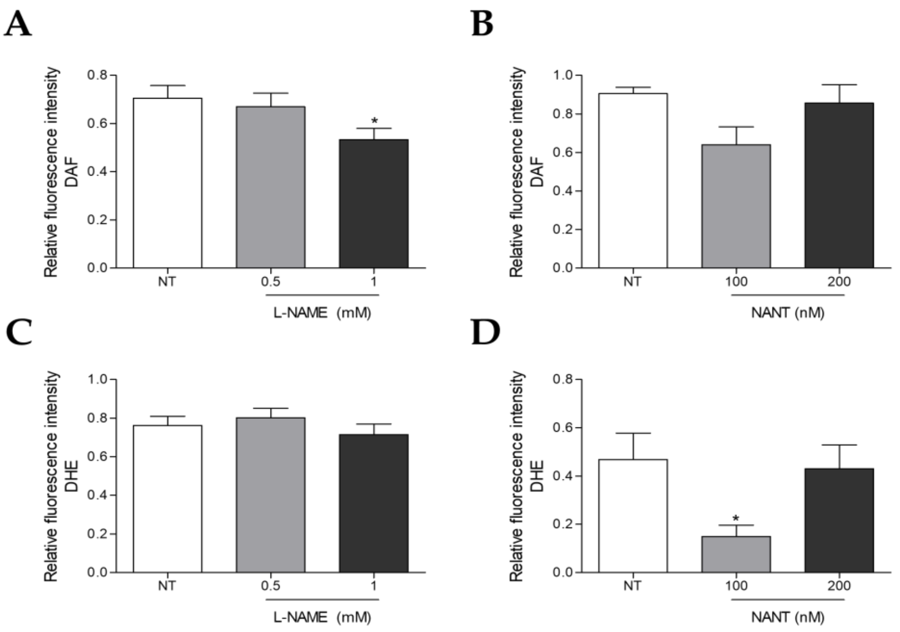

Figure S2. Neuronal nitric oxide synthase is uncoupled in metastatic melanoma. WM983B cells were treated or not for 45 min with 0,5 and 1 mM L-NAME, an eNOS inhibitor **(A, C)** or 100 and 200 nM NANT, a nNOS inhibitor **(B, D)**.NO amount was evaluated by flow cytometry using DAF **(A,B)** and $O_2^{\bullet-}$ levels using DHE **(C,D)**. Values are reported in the bar graphs and expressed as the means ± S.D. The experiments were performed in triplicate and p values were based on One-Way ANOVA test followed by Bonferroni's post-test *p<0.05.

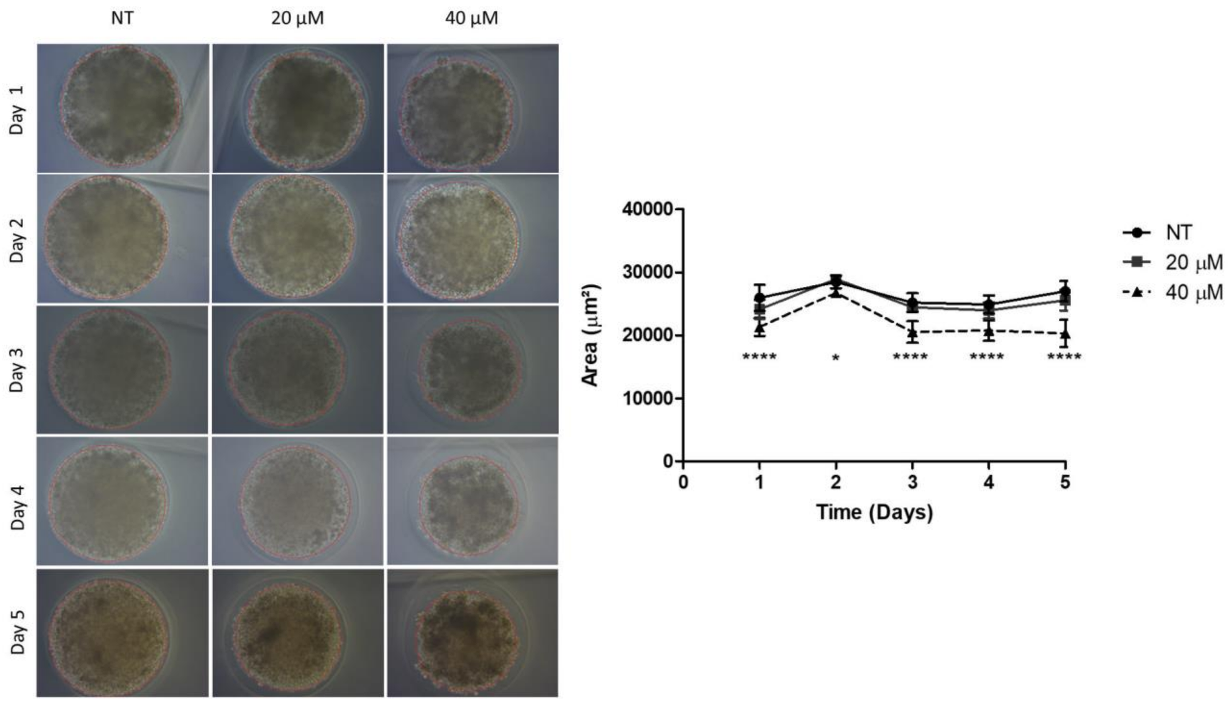

Figure S3. Tumorsphere formation reduction in the presence of BH4. WM1552C RGP melanoma cells was treated or not with 20 and 40 μM with BH4 and tumorsphere area was calculated over five days. Values are reported in the bar graphs and expressed as the means ± S.D and p values were based on Two-Way ANOVA test followed by Bonferroni's post-test , *p<0.05, ***p<0.001.

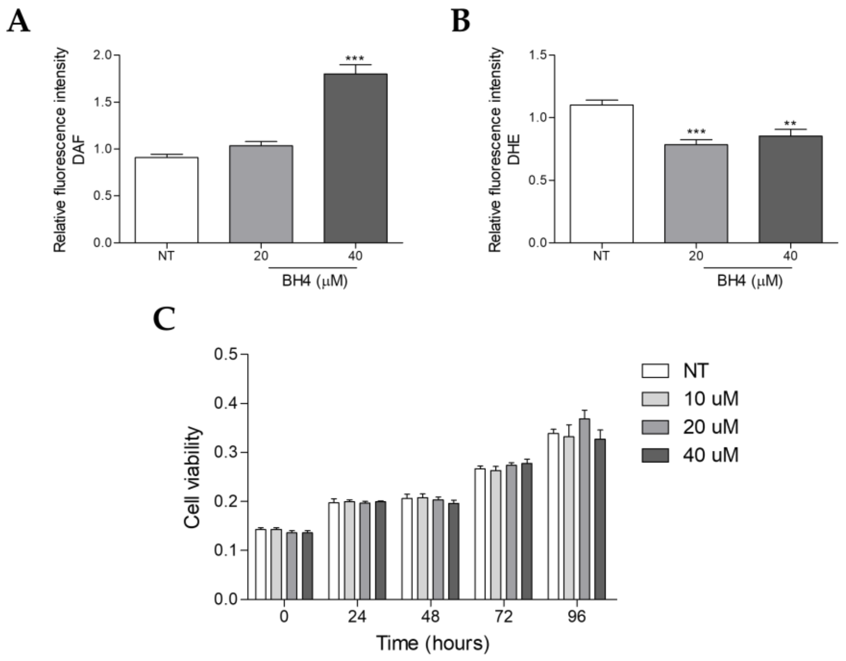

Figure S4. The viability of WM983B melanoma cells was not impaired by tetrahydrobiopterin. WM983B was treated or not with 20 and 40 μM with BH4 and NO amount was evaluated by flow cytometry using DAF **(A)** and $O_2^{\bullet -}$ levels using DHE **(B)** and with 10, 20 and 40 μM BH4 for 24, 48, 72 and 96 hours and cell viability was evaluated by MTT **(C)**. Values are reported in the bar graphs and expressed as the means ± S.D. The experiments **(A,B)** were performed in triplicate and p values were based on One-Way ANOVA test followed by Bonferroni's post-test , **p<0.01, ***p<0.001. MTT analyses were performed in triplicate and p values were based on Two-Way ANOVA test followed by Bonferroni’s post-test *p<0.05.

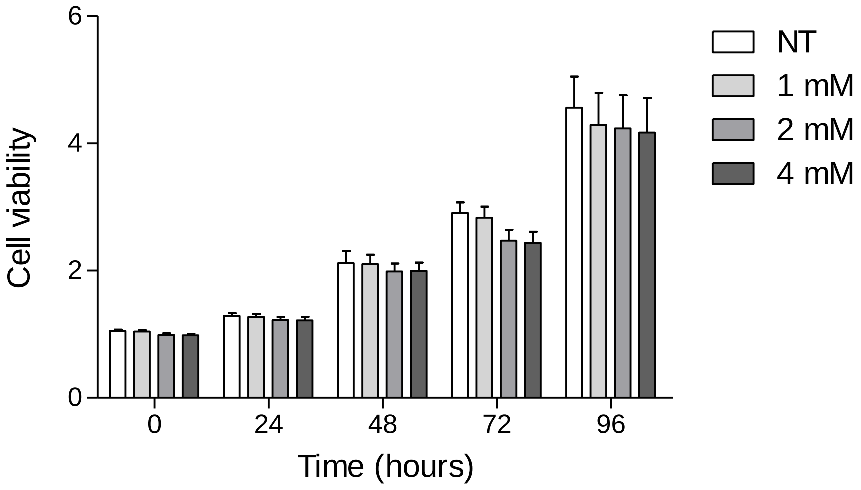

Figure S5. Melanocytes viability is not affect by DAHP treatment. NGM melanoctyes were treated or not with 1, 2 e 3 mM DAHP, the GTPCHI inhibitor, for 24, 48, 72 and 96 hours and cell viability was evaluated by MTT. The experiments were performed in triplicate and p values were based on One-Way ANOVA test followed by Bonferroni's post-test *$p<0.05$.
